# Beriberi Induced Cardiomyopathy Requiring Salvage Venoarterial Extracorporeal Membrane Oxygenation

**DOI:** 10.1155/2016/5043818

**Published:** 2016-12-05

**Authors:** Aditya Shah, Samir Patel, Sorabh Kothari, Jennifer Denk

**Affiliations:** ^1^University of Illinois at Chicago, Chicago, IL, USA; ^2^Advocate Christ Medical Center, Oak Lawn, IL, USA

## Abstract

Beriberi refers to a constellation of symptoms caused primarily by thiamine (vitamin B1) deficiency. An acute and fulminant presentation of this rare condition has been described in the literature as “Shoshin” beriberi which is characterized by catastrophic cardiovascular collapse. Early recognition and treatment lead to dramatic improvements of symptoms. We present a case of thiamine deficiency-induced acute heart failure in a malnourished patient leading to cardiac arrest necessitating VA-ECMO (venoarterial extracorporeal membrane oxygenation) with improvement in heart function secondary to thiamine administration.

## 1. Introduction

Beriberi refers to a constellation of symptoms caused primarily by thiamine (vitamin B1) deficiency. Beriberi has traditionally been divided into four separate entities, relating to the organ system mainly involved (nervous, cardiovascular, or gastrointestinal) with an additional specification for age (i.e., infantile) [1–5]. The neurologic form has traditionally been referred to as “Dry beriberi,” whereas the cardiovascular form is known as “Wet beriberi.” An acute and fulminant presentation of this rare condition has been described in the literature as “Shoshin” beriberi which is characterized by catastrophic cardiovascular collapse [6]. This condition requires early recognition and management, often leading to dramatic improvement in symptoms, whereas failure to diagnose and treat can have devastating consequences.

## 2. Case Report

Our patient was a 33-year-old female who in adolescence and early adulthood was diagnosed with multiple allergies manifesting as erythema, urticaria, and dyspnea. As a result, she consumed an increasingly restrictive diet. Initially, she was diagnosed with a latex allergy; hence she avoided all cross reactants known to be associated with this. At 22 years of age, she was found to have a corn allergy and therefore she eliminated corn and all corn related foods from her diet. Her symptoms subjectively improved after these lifestyle modifications.

5 years before presentation, she experienced unintentional weight loss from her baseline of 61.7 kilograms owing to an extremely restricted diet to a nadir at presentation of 40.8 kilograms. She also endorsed body aches, alopecia, dyspnea, fatigue, headaches, Raynaud's phenomenon, and cold intolerance. She had no pertinent familial, surgical, or social histories.

Our patient's condition declined significantly 1 month before her admission when she was admitted for palpitations, intermittent hematochezia, and worsening shortness of breath. She was found to have dark subcutaneous nodules on her shins, which were diagnosed as erythema nodosum accompanied by Raynaud's phenomenon. She was found to be anemic to 7.3 milligrams/deciliter with leukopenia at 1.9 thousand/microliter. Iron studies yielded mixed results consistent with iron deficiency anemia along with low folate and vitamin B12 levels. For her dyspnea, a contrast enhanced chest computerized tomography scan and lower extremity venous Doppler were done, which were negative with respect to thromboembolic disease. An echocardiogram revealed normal systolic and diastolic function without valvulopathy. Given her leukopenia and anemia, outpatient bone marrow biopsy, esophagogastroduodenoscopy, and colonoscopy were recommended. Celiac disease and autoimmune diagnostics produced negative results. Over the course of the admission, the patient's shortness of breath improved with hemoglobin stabilization.

She was discharged home on ferrous sulfate for her anemia, folic acid supplement, B12 1000 micrograms intramuscularly once a week for four weeks, and scheduled for outpatient follow-up with gastroenterology as well as the hematology service for the diagnosis of mixed anemia.

Approximately 2 weeks after discharge, she represented with shortness of breath, generalized weakness, and pallor. According to the patient's husband, she became debilitated from her dyspnea and generalized weakness which resulted in an inability to walk. In the emergency department, she was afebrile, hypoxic with oxygen saturation of 87 percent on 6-liter nasal cannula, and tachycardic at 120 beats per minute with a blood pressure of 102 millimeters Hg/67 millimeters Hg. On physical exam, the patient appeared in moderate distress, with auscultation of the lungs revealing crackles bilaterally, with bilateral lower extremity subcutaneous nodules and rash.

**Figure 1:**
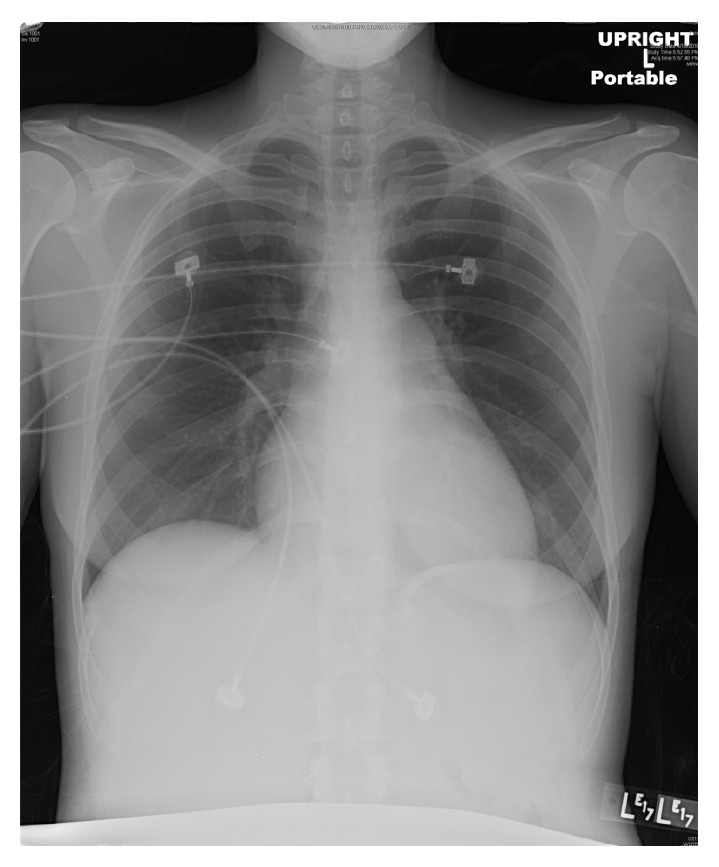
Initial chest X-ray showed no acute findings.

**Figure 2:**
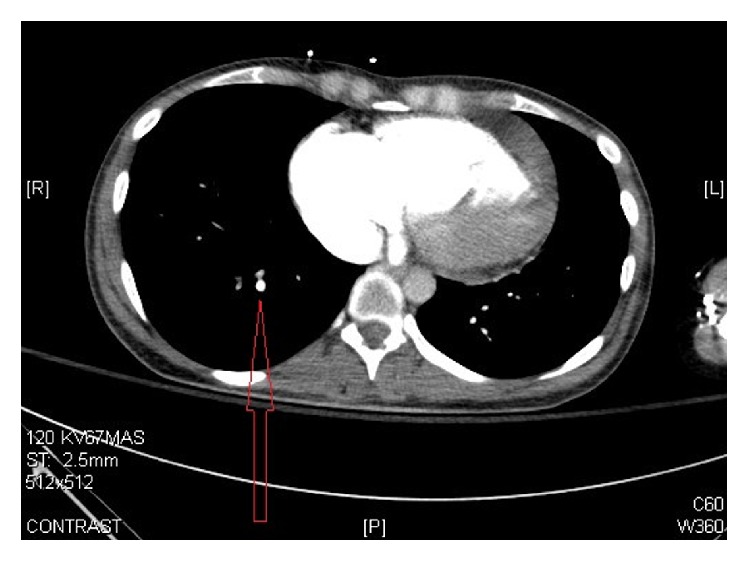
CT scan with contrast of the chest showing small distal right pulmonary embolus.

**Figure 3:**
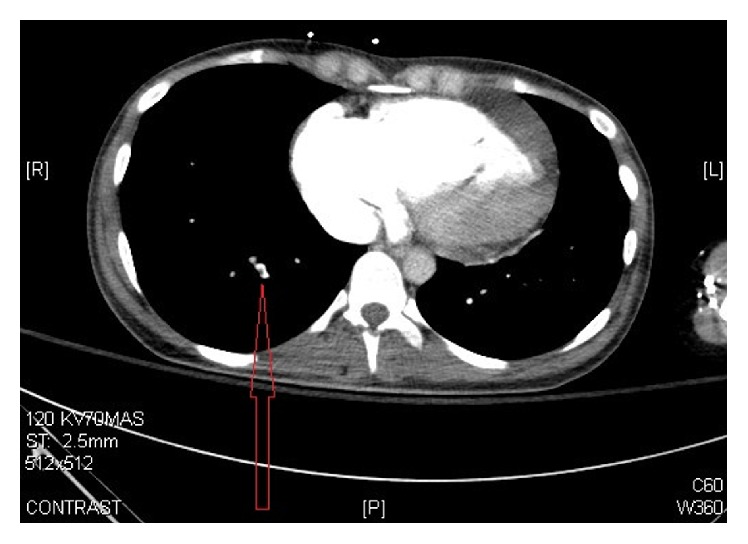
CT scan with contrast of the chest showing small distal right pulmonary embolus.

**Figure 4:**
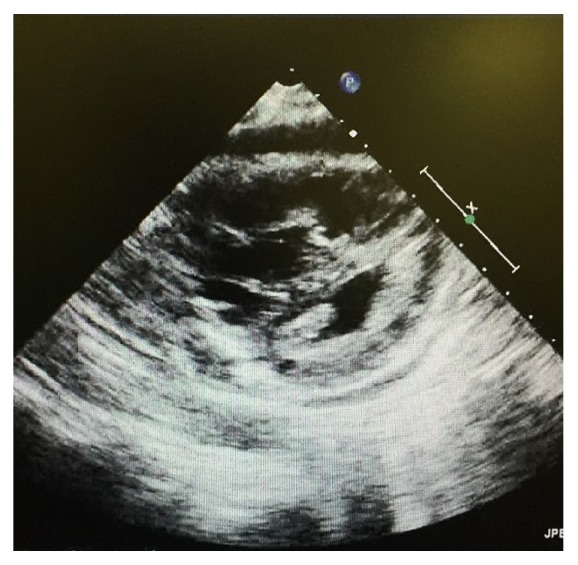
Echocardiogram showing significant dilation of right ventricle along with a slit-like left ventricle.

Chest X-ray initially showed no acute findings (Figure 1). A contrast enhanced chest tomography study was done, which showed a small distal right pulmonary embolus (Figures 2 and 3). An echocardiogram was performed, the parasternal short axis of which showed significant dilation of the right ventricle along with slit-like left ventricle (Figure 4). During positioning of patient to left lateral decubitus position for optimal echocardiographic imaging, the patient sustained a pulseless electrical activity (PEA) cardiac arrest requiring cardiopulmonary resuscitation along with intubation with return of spontaneous circulation after two cycles. She required epinephrine and norepinephrine infusions for hemodynamic support after return of spontaneous circulation.

**Figure 5:**
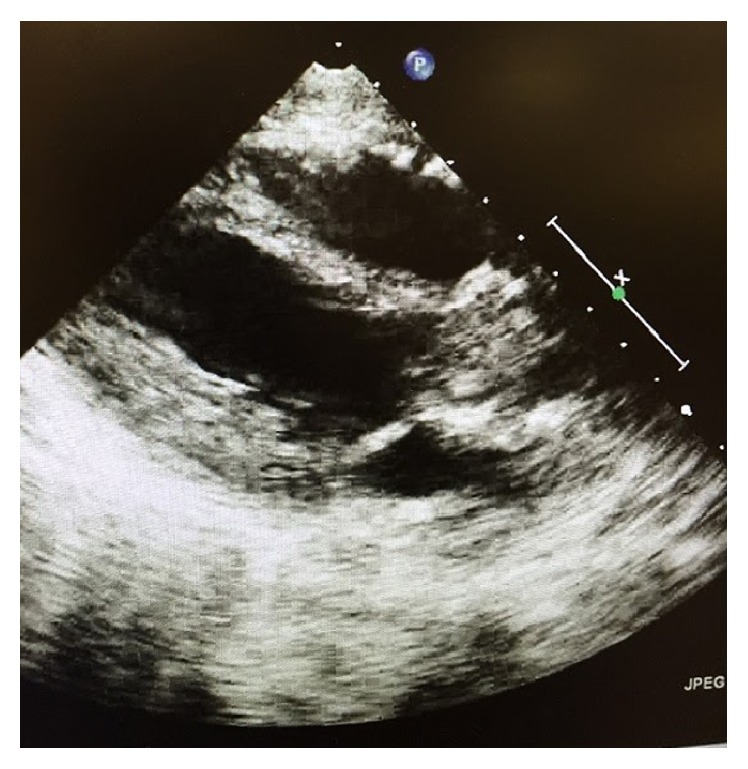
Repeat echocardiogram showing larger left ventricle with markedly reduced function, as well as mitigation of previously overloaded right ventricular size and function.

**Figure 6:**
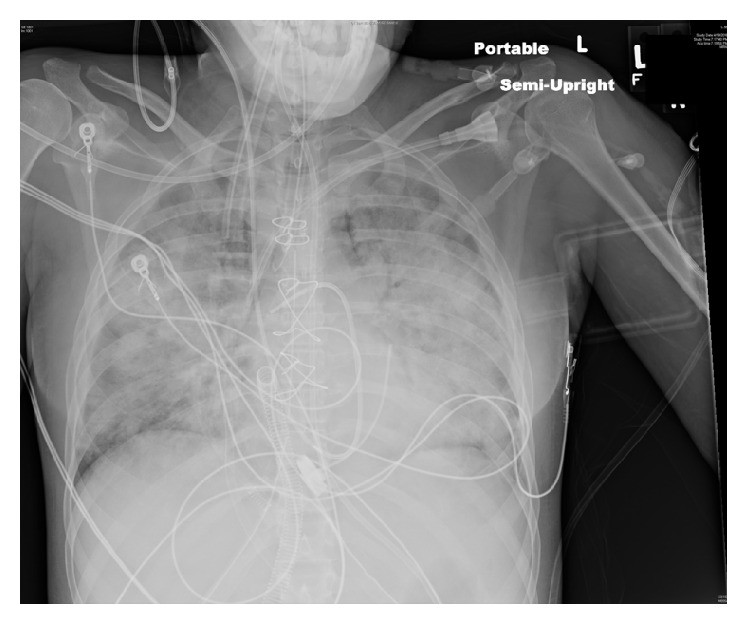
Repeat chest X-ray after intubation shows worsening bilateral aeration with developing patchy infiltrates.

A Swan-Ganz catheter was placed which showed an initial central venous pressure of 11 millimeters Hg, right ventricular systolic pressure of 40 millimeters Hg/18 millimeters Hg, and a pulmonary artery (PA) pressure of 45 millimeters Hg/24 millimeters Hg with a mean pulmonary artery (PA) pressure of 35 millimeters Hg with wedge pressure of 19 millimeters Hg and mixed venous saturation of 60%, cardiac output at 3 liters/minute, and systemic vascular resistance of 500 dynes/second/centimeters^−5^. Repeat echocardiogram was completed, which in the parasternal long axis showed a larger left ventricle with markedly reduced function, as well as mitigation of the previously overloaded right ventricular size and function (Figure 5). A repeat chest X-ray after intubation showed worsening bilateral aeration with developing patchy infiltrates, not seen on previous imaging (Figure 6). The pulmonary artery catheter measurements showed abnormal right and left sided pressures consistent with the dynamic echocardiographic changes noted on serial imaging.

The patient's laboratory values on current presentation showed an elevated B-type natriuretic peptide of 1,602 nanograms/liter which was thought to be secondary to new onset congestive heart failure and elevated aspartate transaminase and alanine transaminase to 1296 units/liter and 977 units/liter, respectively, possibly secondary to ischemic hepatopathy with acute kidney injury noted with a creatinine of 1.18 milligrams/deciliter. Her troponin level was elevated at 0.06 micrograms/liter prior to the cardiac arrest.

With profound hypoxemia and cardiovascular instability after cardiac arrest, the patient was taken to the operating room and placed on central VA-ECMO owing to difficult anatomical peripheral cannulation. She required ECMO support for 14 days prior to decannulation before requiring tracheostomy. Her hospital course was further complicated by compartment syndrome of the left lower extremity requiring emergent bedside fasciotomy as well as arterial thrombus extending from the descending aorta to the superior mesenteric artery for which she underwent open surgical thrombectomy. The patient required continuous renal replacement therapy (CRRT) for oliguric renal failure along with cholecystostomy tube for acalculous cholecystitis.

An extensive workup was performed due to her complicated presentation and severity of illness. Given the patient's poor nutritional status as well as persistent lactic acidosis, a vitamin B1 (thiamine) level was obtained with both plasma and whole blood levels. The plasma level was remarkably low, <2 nanomoles/liter (reference range: 8 to 30 nanomoles/liter), and her vitamin B1 whole blood level was 91 nanomoles/liter (70 to 180 nanomoles/liter). Her vitamin B6 was also low, 15.2 nanomoles/liter (20 to 125 nanomoles/liter).

**Figure 7:**
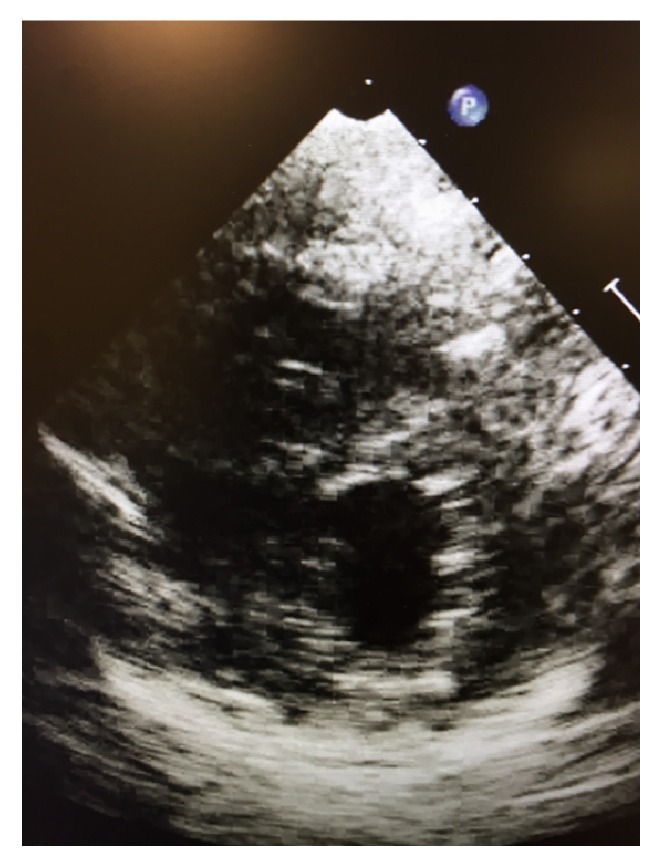
Echocardiogram parasternal short axis showing left ventricular chamber back to normal size and function.

The patient was initiated on intravenous thiamine prior to laboratory confirmation of low plasma levels. Over the course of her stay, the patient showed significant improvement in her respiratory, cardiovascular, and renal status and was eventually decannulated from VA-ECMO after 14 days with transition to mechanical ventilation and eventual tracheostomy. Her cardiovascular function showed marked improvement both clinically and echocardiographically concurrent with thiamine administration. Three days after initiation of thiamine treatment, her ejection fraction, initially noted at 20 percent while on vasopressors, showed serial increases to 50% and then six days later to 70% (Figure 7). The patient no longer required inotropic support and was weaned off vasopressors. The patient was discharged from the intensive care unit neurologically intact to a telemetry floor and subsequent inpatient rehab after which she was discharged home.

## 3. Discussion

Beriberi is a rare condition in developed countries [7]. “Shoshin” beriberi is an even less common condition. The diagnosis depends on clinical manifestations of heart failure with the exclusion of other traditional etiologies and prompt response to therapy. With adequate clinical suspicion, the clinical findings can fit a classically pathognomonic picture [8, 9]. Assistance is often needed with additional imaging and lab studies to confirm clinical suspicion.

Transthoracic echocardiography can be used to aid in the diagnosis of this rare condition. Classic echocardiographic findings for cardiac beriberi could include left ventricular enlargement with or without reduced function and can also be accompanied by valvular dysfunction. Empiric therapy with thiamine could be a reasonable option owing to the nonspecific cardiac imaging findings and lack of real-time lab data [10]. In addition, there is virtually no downside to empiric treatment with thiamine in patients with normal renal function as the kidneys can rapidly clear excess thiamine.

Our patient is unique given that she had biventricular failure with dynamic echocardiographic changes necessitating VA-ECMO for profound hemodynamic collapse. Given the stark contrast with recent echocardiographic results, we hypothesize that the eventual cardiac dysfunction initially undetected could have been sequelae of her worsening clinical condition with a nonexistent cardiac reserve secondary to thiamine deficiency. The subsequent hospital course with infection and embolic and thrombotic phenomena were thought to be secondary to mechanical complications of extracorporeal membrane oxygenation as these issues were mitigated with decannulation.

A contrast enhanced chest tomography study done on presentation to the emergency room showed an incidental small distal pulmonary embolus with initial right ventricular strain pattern on echocardiogram. We surmise that the patient had decompensated heart failure on presentation as evidenced by elevated beta natriuretic peptide, transaminitis, and hypoxia. Given her triad of lactic acidosis and hypoxia with confounding pulmonary embolism, her pulmonary vascular resistance increased causing right sided heart failure with subsequent cardiac arrest.

We underscore the importance of serial echocardiography in acutely ill patients. Given her CT evidence of thromboembolic disease with initial echocardiographic images showing severe right ventricular strain, thrombolytic therapy was initially entertained given the clinical picture but repeat echocardiography led to reconsideration of this therapy with VA-ECMO opted for emergently. Given her central cannulation and significant transfusion requirement intraoperatively, thrombolytic therapy for pulmonary embolism quite possibly could have resulted in disastrous consequences.

Beriberi frequently is a diagnosis of exclusion. The patient's initial presentation is unique given her dynamic echocardiographic changes and concurrent pulmonary embolism with initial mixed venous saturation pointing against high output heart failure. We postulate that her venous saturation and cardiac output may have been underestimated given her profound anemia noted previously along with compromised right ventricular output owing to her significant acidosis and hypoxia. Her systemic vascular resistance was noted to be low, a finding unexpected in cardiogenic shock. We attribute this to the direct effects of thiamine deficiency on vasomotor tone similar to previous reports [11].

The patient received thiamine intravenously with rapid improvement in her cardiac hemodynamics on subsequent echocardiographic studies. The patient is currently doing well and has been discharged home after an acute rehabilitation stay of two weeks.

## 4. Conclusion

Beriberi induced cardiomyopathy is a rare occurrence in developed countries. To our knowledge, there is a paucity of literature supporting VA-ECMO (venoarterial extracorporeal membrane oxygenation) as a bridge while treating cardiac beriberi. We stress the importance in maintaining a high index of suspicion for beriberi with new onset heart failure in a malnourished young patient.
